# The devil is in the task structure

**DOI:** 10.7554/eLife.78930

**Published:** 2022-04-27

**Authors:** Orit Nafcha, Uri Hertz

**Affiliations:** 1 School of Psychological Sciences, University of Haifa Haifa Israel; 2 Department of Cognitive Sciences, University of Haifa Haifa Israel

**Keywords:** social learning, dopamine, reinforcement learning, reward learning, haloperidol, learning, Human

## Abstract

Conflicting evidence about how the brain processes social and individual learning stems from which type of information is presented as the primary source of knowledge during experiments.

**Related research article** Rybicki AJ, Sowden SL, Schuster B, Cook JL. 2022. Dopaminergic challenge dissociates learning from primary versus secondary sources of information. *eLife* **11**:e74893. doi: 10.7554/eLife.74893.

How should you pick your next read? You may rely on the recommendations from a renowned critic, or base your selection on specific literary genres. If you enjoy the book you chose, you will then update how you evaluate these sources of information: maybe historical fiction really is to your taste, and maybe you will keep on following that particularly perceptive critic. In many cases these two ways to acquire and assess information – one social and relying on the critic, the other individual and based on the characteristic of the item – occur at the same time (Figure 1A).

**Figure 1.**
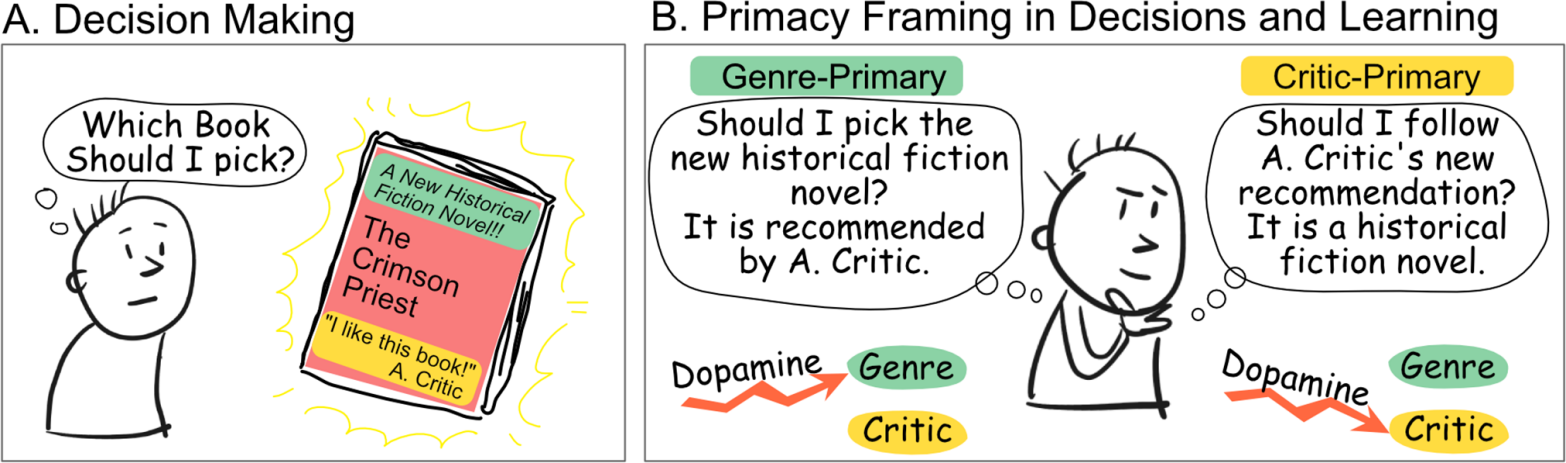
Anatomy of a decision-making process based on multiple types of information. (**A**) An agent can use multiple sources of information when making decisions, for instance, which book to read next. In that example, they can use item-based information, such as the genre of the book (green), or social-based information, such as whether it has been recommended by a well-known critic (yellow). Feedback (Was the critic right? Was that specific genre enjoyable?) can drive learning about both sources of information. (**B**) The way the decision is framed and presented could make one source of information the primary source, and the other the secondary or supporting source. Results from Rybicki et al., 2022 suggest that the primacy of the source of information, and not its type (social/item-based), determines its dependency on dopaminergic learning mechanisms.

In social learning, knowledge about the world is acquired by observing or interacting with another agent (Olsson et al., 2020). However, during individual learning – when decisions are based solely on the features of an object – the learner obtains knowledge directly, through trial-and-error. But do social and individual learning rely on two different neural mechanisms, or are they just two different sources of information?

So far, existing research presents mixed evidence. Some studies find that in dual learning tasks – when a participant gathers information through both social and individual learning – the two types of knowledge acquisition elicit distinct brain activity (Behrens et al., 2008; Diaconescu et al., 2020; Zhang and Gläscher, 2020). Other experiments, however, suggest that there is no neural mechanism specific to social learning. For example, previous work has revealed that individual and social learning compute information similarly, calculating the mismatch between predicted and experienced outcomes; and that being forced to primarily use social information for a decision triggers activity in dopamine-rich brain regions previously linked to individual learning (Diaconescu et al., 2017; Olsson et al., 2020; Iglesias et al., 2013). Now in eLife, Alicia Ribicki, Sophie Sowden, Bianca Schuster and Jennifer Cook from the University of Birmingham report that the structure of the experimental task, rather than the type of learning, is responsible for these mixed results (Rybicki et al., 2022).

In the most popular version of the dual learning task, participants are asked to decide between two options (for instance, whether a reward is hidden in a blue or a green box) while also receiving a hint from a social agent - ‘an adviser’ (Behrens et al., 2008). When the outcome of the choice is revealed (was the reward in the chosen box?), participants can update their evaluation of the boxes’ values, and infer the accuracy of the adviser. However, Rybicki et al. note that this version of the task forces participants to mostly rely on information relevant for individual learning; they therefore label this condition ‘Individual-Primary’, where ‘primary’ refers to information that appears first, is highly salient and is directly related to both making and assessing a decision. The team hypothesized that differences in how the brain processes social and individual learning during this task may be related to the fact that the design confounds the type of learning (social vs. individual) and the primacy of the information source (primary vs. secondary).

To test this, Rybicki et al. developed a new task which included both the classic Individual-Primary condition and a new, ‘Social-Primary’ condition during which participants use the adviser’s recommendation as the primary source of information, along with secondary information about the recommended boxes. When the outcome of the choice is revealed, volunteers can update their evaluation of the adviser’s accuracy (did the recommendation lead to finding the reward?) and infer the value of the boxes. Participants in both the Individual-Primary and the Social-Primary conditions also performed the task twice: under a placebo, or under a drug – haloperidol – that perturbs dopamine-based signaling in the brain (Schultz, 2007). This allowed the team to dissect the role of dopamine in the learning process.

The results showed that dopaminergic manipulation affected the type of learning (social vs. individual) when that specific type was the primary but not the secondary source of information (Figure 1B). Previous findings demonstrating dopamine-dependent learning for individual but not social learning may therefore be related to the fact that individual learning, in these experiments, was the primary way to obtain information. This also explains why dopaminergic-dependent learning was observed in both a social and individual context in tasks with only one source of information. This finding may have a profound impact on the ongoing debate about how the brain processes social and individual information, shifting the attention to the task structure and away from the type of learning. Concrete health implications may emerge as task structure, and not content, may be the basis of psychiatric conditions and symptoms such as paranoia (Reed et al., 2020).

When creating the new version of the task, Rybicki et al. manipulated primacy by simultaneously adjusting several variables, such as the saliency of the signal, when it appeared, and the type of choice and reward feedback. What exactly makes a source of information ‘the’ primary signal therefore remains an open question. It is possible, for instance, that information which allows learning by directly linking the choice with the outcome (whether the right call was made) is used primarily. Secondary information would require an extra step where the participant, for example, must infer that the recommendation was accurate because the outcome was good. However, Diaconescu et al., 2017 showed that when there is only one source of information, even inference-based learning can be dopaminergic related. More work is needed to determine how task properties relate to neural learning mechanisms.

The study by Rybicki et al. has important implications for the way that social learning is studied and understood. If social and individual learning rely on the same neural processes, then what is already known about learning in a non-social context – the neural and computational mechanisms involved in processing that information, for example – could then be deployed in the social domain (Lockwood et al., 2020). On the other hand, knowing what the two types of learning share could also help to dissect the unique contribution of the social world to learning (Heyes, 2012). For example, the fact that various social traits such as prosociality and selfishness may be processed differently could be attributed to the unique characteristics of these social inputs, rather than to the learning process itself (Siegel et al., 2018). Delineating what makes social learning special while acknowledging that it may be based on a domain-general learning mechanism may lead to a richer understanding of the way the social environment shapes cognition.
